# NEO-SPEAK: A conceptual framework that underpins breaking bad news in neonatology

**DOI:** 10.3389/fped.2022.1044210

**Published:** 2022-11-09

**Authors:** Carola Seifart, Mirjam Falch, Mirjam Wege, Rolf F. Maier, Anna J. Pedrosa Carrasco

**Affiliations:** ^1^ Faculty of Medicine, Dean’s Office, Research Group Medical Ethics (AGEM), Philipps-University of Marburg, Marburg, Germany; ^2^ Children’s Hospital, University Hospital of Marburg, Marburg, Germany; ^3^ Faculty of Medicine, Philipps University of Marburg, Marburg, Germany

**Keywords:** breaking bad news, communication, parent-physician relationship, communication barriers, neonatal intensive care, professional-family relations, truth disclosure

## Abstract

**Objective:**

Breaking bad news in neonatology is a frequent and difficult challenge. Although there are guidelines for communicating with parents in pediatrics and neonatology, the specific framework for breaking bad news in neonatology has not been studied in more detail. Therefore, we aimed to identify determinants that are important for successful managing breaking bad news in neonatology from professionals' perspective and to develop a conceptual framework that underpins this challenging task.

**Methods:**

We conducted seventeen semi-structured interviews with senior neonatologists of six perinatal centers of the highest level of care in Germany. The transcripts were analyzed according to Mayring's method of qualitative content analysis using inductive and deductive coding.

**Results:**

Eight determinants of breaking bad news in neonatology could be identified from the interviews. From these, we developed the conceptual framework NEO-SPEAK. The first three determinants, Neonatal prognostic uncertainty, Encounter in (triangular-)partnerships, Organization and teamwork (NEO) are directly related to the specific care situation in neonatology, whereas the others, Situational stress, Processuality, Emotional burden, Attention to individuality, Knowledge and experience, play a role for difficult conversations in general, but are subject to special modifications in neonatology (SPEAK). In addition, the results show that the context in neonatology as well as reciprocal effects on the team and the individual level of the physicians are important influencing factors in breaking bad news.

**Conclusion:**

On the one hand, the constitutional framework NEO-SPEAK shows which special aspects play a role in neonatology for the delivery of bad news, and on the other hand, it can help to identify and consider these aspects in clinical routine and training. Considering or reinforcing each NEO-SPEAK element when planning or delivering bad news may guide healthcare professionals through communication with parents of critically ill or premature newborns and support the resilience of the caring team.

## Introduction

About one in 16 neonates spends some time in a neonatal intensive care unit (NICU) (1), not surprisingly the risk increases as gestational age decreases (2). According to the World Health Organization, each year about 15 million babies are born prematurely, i.e., before 37 weeks of gestational age (3). Although in Germany survival rate of infants with very low birth weight (below 1,500 g) exceeds 90 percent (4), preterm birth remains a significant risk factor for infant mortality and child morbidity and disability (5). Diagnoses leading to NICU admission are manifold and include, in addition to prematurity, respiratory, cardio-circulatory, gastrointestinal, neurological, and infectious problems that can have a long-lasting impact on the child's health (6–9).

Parents of newborns needing intensive care are heavily burdened by their infant's medical condition. Many studies report the NICU admission to be a stressful experience for parents (10–14) compromising their mental and emotional health (13, 15–18). They can be faced with feelings of loss of control, restlessness, anxiety, and fear (14, 19), as well as post-traumatic stress reactions even with short NICU stays (20, 21). In these situations, neonatologists need to regularly exchange information with parents about the infant's condition and prognosis, including giving bad news. Bad news is defined as “any information that produces a negative alteration to a person's expectations about their present and future” (22). It is known, that the process of breaking bad news is an important determinant for the psychological well-being of the recipients in general (23, 24). It is thus all the more alarming that there are reports on persisting shortcomings in parent-staff interaction in the NICU (25). Unfortunately, neonatologists' communication skills and priorities during consultations as well as a lack of time do not always meet the parents' needs (26–29), whereas balanced communication directly influences parents' confidence and reassurance as well as successful parent-child bonding (13, 17).

True, there are guidelines for communicating with parents in pediatrics and neonatology, and special etiquette has been proposed (30). But as far as we know, elementary principles and challenges in the transmission of bad news in neonatology have not yet been explored in detail. However, additional information about the underpinning framework on breaking bad news in neonatal intensive care would be beneficial for two reasons: Better understanding of the context and the precondition for delivery of bad news could (i) improve patient- and family-relevant outcomes but also (ii) reduce a well-known source of moral and emotional distress in NICU team members (31, 32).

Aware of the lack of sound knowledge about delivering bad news in the NICU and its influence on parents' and healthcare professionals' personhood (33–36), we conducted a qualitative study exploring neonatologists' point of view on successfully managing breaking bad news-discussions in the NICU. By analyzing the important determinants, we aimed to derive a theoretical framework for the essential foundations and potential difficulties of communicating troubling news in neonatology.

## Methods

We conducted audio-recorded interviews with 17 consultants from six different perinatal centers of the highest level of care in Germany. All participants were pediatricians with additional specialist training in neonatology. Thus, senior clinicians were included who could be expected to have a wealth of experience in neonatal intensive care. We recruited participants through personal contact (RFM). Purposive sampling was used in terms of age, gender and hospital location and characteristics to capture a breadth of views and experiences among the study participants.

The topic guide was developed by a multi-professional team (MF, MW, RFM, CS) with expertise in neonatology, intensive care, psychology and ethics. It included open-ended questions about personal experiences with breaking bad news, factors that influence the effective delivery of bad news, and developments or circumstances in the health system that could potentially have an impact. Before the interviews began, it was indicated that two angles, namely the expert-on-expert and expert-on-parent perspectives, were of particular interest for the research question.

Following written informed consent, semi-structured face-to-face interviews were conducted by MF until the research team agreed that data saturation was achieved. Interviews were digitally audiotaped. Additionally, reflective field notes were written by MF immediately after each interview and integrated into the analysis. All interviews were transcribed verbatim and identifiable references were anonymized. MAX-QDA 12 (Release 12.3.2) was used for data management. The analysis process was based on Mayring's qualitative content analysis (37). A deductive-inductive coding approach was chosen resulting in a refined code structure by comparing codes both within and across interviews identifying overarching themes. The deductive structure was grounded in the SPIKES protocol (32). Coding was carried out independently by two different researchers (MF, MW) but the analysis progress was critically reviewed and discussed in regular meetings of the larger research group to enhance rigor and confirmability. The qualitative results were ultimately synthesized by the research group into a conceptual framework. All interviews were performed in German but translated into English for publication by a fluent native speaker.

Ethical approval was obtained from the institutional review board of the Philipps-University Marburg (ID-No.: 105/15). The study was conducted in accordance with the Declaration of Helsinki, Good Clinical Practice guidelines, and the EU data collection directive.

## Results

We performed interviews with 6 female and 11 male senior neonatologists between November 2015 and July 2017. Participants' mean age was 48 years (SD 9 years, range 36–62 years).

Across all interviews, from the participants' point of view, successful managing of difficult conversations in neonatology demands consideration of the special neonatological context, interaction levels and organizational requirements. Conversations of bad news should be context-sensitive, thus adapted to the situation at hand, always reflecting the special situation in neonatology. The interaction levels in neonatology are particularly complex due to triangular relationships and specific care structures. An intensive interplay between parents and team members was described, in the context of which attention must be paid to the individual stakeholders. While acknowledging that parents are indisputably exposed to a great psychological burden, the difficult task of breaking bad news also leads to an emotional strain on the team, particularly on the members delivering the news. The stressful situations in which both parties find themselves in turn influence the conversation process in a sustained and reciprocal way.

Specifically, we identified eight interrelated determinants underpinned breaking bad news in neonatology: neonatal prognostic uncertainty, encounter in (triangular-)partnerships, organization and teamwork, situational stress, processuality, emotional burden, attention to individuality, knowledge and experience. From these we developed the conceptual framework NEO-SPEAK.

### N-E-O

The three determinants neonatal prognostic uncertainty, specific partnerships and organizational aspects could be grouped under neonatal context sensitivity as each refers to common clinical reality in neonatology. All three determinants are closely interwoven and affect each other.

#### Neonatal prognostic uncertainty

Neonatology is a special field of pediatrics dealing with the typical diseases of newborns and the special needs of premature babies. The neonatologists deemed the diagnosis and the resulting prognosis to be a natural determinant of informative conversations with parents. Participants reported higher stress levels when sharing unfavorable diagnoses. However, in neonatology, characteristically a high level of prognostic uncertainty plays a crucial role, which clearly complicates and hamper conversation processes.

*“With asphyxia it is similar […] when in the end you can only talk about the risks. But I always find it easier for such conversations when it is a clear situation. Even if it's just…Even if it's catastrophic, it's catastrophic, but catastrophic and clear, so to speak. And then when it's vague, then it's sometimes difficult, so to speak, then it also very much depends on the other person.” (Expert D, male, 39 years)*

#### Encounter in (triangular-)partnerships

As usual in pediatrics, the network of relationships differs from other medical disciplines in neonatology. First, there is a triangular relationship between parents, patients and physicians.

*“But, thank God, there are the majority of conversations that enable a common ground, a common basis for further action on the infant or the accompaniment/care of the infant and create a basis of trust. And that is actually what we want to achieve, that despite a difficult prognosis, perhaps a bad prognosis, we can move forward together with the parents.” (Expert C, male, 62 years)*

Second, related to the specific situation of medical care, there is more triangular relationship between parents, nurses and physicians. These pre-conditions influence the conversation process relevant.

*“It should be an assessment of the team. It shouldn't be judged by one this way, and that way by another person, and everyone talks to the parents as they personally experience it. Yes, that would be - that is a disaster!” (Expert R, female, 55 years)*

Encounter in partnership in breaking bad news-discussions with parents refer to mutual trust, openness, dealing with emotions and ultimately shared decision-making. During the breaking bad news-process, participants conceded to both professionals and parents a range of feelings that influence discussions. For example, the uncertainty often experienced (3.1.1.) about the diagnosis can lead to hope in some or confirm fears in others, which in return are projected onto the physician.

*“Of course, you feel more positive about yourself when they become sad and not aggressive, but sometimes, of course, aggression is a reaction.” (Expert K, female 57 years)*

#### Organization and teamwork

The neonatologists interviewed stated that the team dynamics may be influential in difficult discussions with parents. Support was perceived through shared decision-making and a positive working atmosphere among team members. Because of the specific situation of prognostic uncertainty and the exceptional importance of informational exchange with caring nurses, it was found crucial to discuss different recommendations for action within the multi-professional NICU team before meetings with parents were held. According to the participants, reaching consensus within the team influence the difficult conversation process and avoid divergent recommendations to the parents and associated burden on family and staff.

*“And in the end, it is also very helpful for the team to have had such a conversation together. There is always mistrust, especially between nurses and doctors, when the doctors have had a conversation […] And if they were there, then it's obvious. So it's good for the team spirit to do it together.” (Expert B, male, 47 years)*

Besides internal team dynamics, political decisions and resulting hospital policies were perceived to adversely affect NICU teams' daily work routine both through the creation of rigid working conditions and additional workload on individuals with noticeable impact on breaking bad news-discussions.

*“Well, it's [workload] already a disaster and it's getting worse. It is absolutely unbearable as it is now…And that is something where I clearly notice that this has an effect on the quality of care, not only on communication but also on medical care.” (Expert B, male, 47 years)*

### S-P-E-A-K

The determinants situational stress, processuality, emotional burden, attention to individuality, knowledge and experience are more principle for difficult conversations in a general sense. However, they are subject to specific conditions in neonatology.

#### Situational stress

The neonatologists recognized the extraordinary situation in the NICU and the associated impact on the parents. The reality of neonatal intensive care often entails the challenge of separation of parents and their infant. In extreme cases, the gestational parent and infant are treated as patients at the same time, and sometimes even have to spend the first few days apart for organizational reasons. For these scenarios, which are often unexpected by parents, participants called for a sensitive approach to breaking bad news. Clinical situations were portrayed in which parents were overwhelmed by events, particularly in acute emergency situations requiring rapid action without room for immediate attention to the parents’ emotions.

*“We then go there as a team and fetch the infant - stabilize it on site if it works and then take the child with us. This means that the initial contact with the parents may be extremely short […] It's really a matter of briefly informing the parents about the situation and nothing more. Which is, of course, quite a slap in the face for the parents.” (Expert Q, male, 36 years)*

#### Processuality

Contrary to the common understanding that an educational conversation refers to a single meeting, the neonatologists interviewed understood the transmission of troubling news as a continuous process. They pointed out that in the NICU setting, the facts to be discussed often have to be obtained over time while the newborńs development has to be observed.

*“That was a successful conversation. But it was not one conversation, it was many conversations, conversations that took place every two days.” (Expert R, female, 55 years)*

Such a communication style was assumed to be responsive to parents' cognitions and emotions. It was thought to have the effect of repeatedly responding to the parents' need for information at an appropriate pace in order to improve their understanding of the clinical situation and to gain support for the proposed treatment options. The participants advised ensuring a constant medical counterpart and firm conditions for follow-up conversations such as fixed appointments. Neonatologists felt that a thereby grown relationship between parents and attending physician at best strengthens trust and helps to cope with possible complications.

*“And after the conversation, the important thing is that there is still a level of trust on which we can continue to work and the parents know: OK, this is the contact person for me now and if I have questions, I can always turn to him or her again.” (Expert M, male, 43 years)*

#### Emotional burden

The participants recognized that parents are exposed to enormous psychological stress, which is influenced by information processing and decision-making. The parents' emotional state was thus seen as an important factor influencing the course of discussions and the parent-staff-relationship [see Section “Encounter in (triangular-)partnerships”]. In addition to the undoubted burden on NICU patients' family members with long-term effects, breaking bad news-conversations have a direct impact on physicians' own emotional state. Participants reported feeling responsible for burdening the recipient. Some admitted to experiencing torment from the task of delivering bad news, at times moving close to feelings of co-causation, complicity, and guilt.

*“What's hard for me - is to take away the parents' hope. There are diseases where you have to say - so (…) according to common sense there is no hope for a halfway normal life. So it is more than difficult for me.” (Expert P, male, 42 years)*

This seemed to translate into active attempts of empowering parents to cope as best as they could with uncertain situations. Most neonatologists did not consider their personal sentiments to be paramount to effective breaking bad news reflecting (i) a professional attitude by primarily focusing on the parents' well-being but also (ii) a tendency to underestimate their own mental hygiene. Despite tense situations in difficult breaking bad news-processes, this behavior suggests the perceived need to put one's own feelings on hold while breaking bad news in order to focus on the parents.

*“And afterwards, the parents said so, so gratefully that it had seemed so calm that I thought: it was pure acting. I wasn't calm at all, I was totally seething inside, but I managed to hide it.” (Expert B, male, 47 years)*

#### Attention to individuality

On the one hand, the theme of individuality aims at the uniqueness of the recipient's personality and personal background, on the other hand, it recognizes the message bearer as an individual, including his or her self-conception.

The study participants emphasized differences in parents' perceptions of situations and subsequent responses, including the ability to be involved in treatment and care decisions for the infant. In their view, diversities in personality, culture, ethnicity, family background and educational level in breaking bad news-discussions should be taken into account. They, therefore, advocated for flexibly addressing the parents' individual needs during each conversation.

*“You can't give a blanket answer because I think all the parents we look after are individually different and every couple needs something different and one of the arts of conversation is to find out what the individual parents need […] There's no one-size-fits-all answer.” (Expert M, male, 43 years)*

For the majority of the neonatologists interviewed, the significance of breaking bad news, was a vital and integral aspect of their medical profession and thus a compelling component of their professional self-concept.

*“Well, this [breaking bad news] is actually the primary medical activity, I think.” (Expert Q, male, 58 years)*

The task of delivering bad news was perceived with a sense of implicitness and responsibility due to clinical routine. In this context, the participants considered their communication skills as an essential determinant for a smooth conversation process

*“A conversation is communication. So if what you want to say to the parents comes across, you notice that. (…) As a doctor, you are the one who shapes the conversation. That means that the doctor basically has it in his hands. So if a conversation fails, it is rather the doctor's fault than the parents.” (Expert L, male, 58 years)*

In addition to the self-concept as a member of a NICU team, the interviewed physicians mentioned their private selves as a determinant for conversation processes when disclosing troubling news, which can conflict with their professional role. Discrepancies can exacerbate physicians' own psychological distress.

*“As I said, it's only difficult when the decision goes against one's own convictions.” (Expert D, male, 39 years)*

#### Knowledge and experience

The neonatologists showed a high level of theoretical expertise in important communication domains including conversation techniques and advantageous conditions in terms of timing, atmosphere, place as well as company. Emotional intelligence, in a sense of perception, understanding, and mindful handling of one's own and others' feelings was seen as a prerequisite for sensitively accompanying the breaking bad news-process. Mindful language use was considered important by the participants and was characterized for them by a respectful, empathetic and at the same time precise, understandable choice of words. Non-verbal communication, especially body language, undivided attention, active listening and mirrored emotions were considered essential. The use of trained and certified non-family translators was recommended for interaction with parents lacking German language skills. The desired mutual trust was regarded to be positively influenced by these competences and transparent information flow but to be impaired by misunderstandings or deviant opinions within the healthcare team. The physicians were aware of the extraordinary situation that parents of unwell newborns may find themselves in. Moreover, they understood potential implications of their statements and comments within discussions.

*“…but if it's only 10% of the words that the parents remember, they remember them for the rest of their lives. […] As a matter of principle, no word should be used superfluously or carelessly in the breaking bad news conversation. So therefore, a certain phrasing is for sure sometimes more delicate than one thinks.” (Expert P, male, 42 years)*

Participants highlighted the significance of structured conversational training, which they felt should begin in the early stages of a physician's careers.

*“Conversational training should be mandatory for all students, not voluntary.” (Expert G, female, 54 years)*

However, there was also agreement that real-life experiences are imperative for effective breaking bad news-conversations.

*“My experience is that you need a lot of experience for informative discussions with parents.” (Expert H, male, 61 years)*

From the participants' point of view, political developments have had an impact on the workload and thus training of resident physicians in the area of communication skills. Increasing staff shortages were blamed for the fact that resident physicians often lacked the opportunity to attend long and challenging conversations led by consultants and are thus deprived of the opportunity to learn by example.

*“…the junior doctor doesn't have time to join in, and that's something that would certainly be important from a training point of view, so to speak, to simply attend a few such conversations” (Expert D, male, 39 years)*

## Discussion

Guidelines and end-of-life care strategies for neonatal intensive care have emphasized the importance of clear, timely, compassionate, and trust-based staff-parent communication (38, 39). However, the literature gives little guidance on how this should be realized in clinical practice and about the factors that set the framework for these difficult conversations. Known frameworks from adult medicine, such as the SPIKES protocol (40), cannot be transferred without further ado, because some conditions and prerequisites are clearly different in neonatology. Therefore, we aimed to analyze the constituent framework determinants for delivery of bad news in neonatology. The analysis of our qualitative interviews shows that, from the neonatologists' point of view, breaking bad news is shaped by eight determinants that are closely interwoven. We have grouped these determinants under the acronym NEO-SPEAK. Table 1 presents the single elements of the conceptual framework and corresponding explanations in detail.

**Table 1:** The conceptual framework NEO-SPEAK for breaking bad news in neonatology.

Initials	Domain	Clarification
N	Neonatal prognostic uncertainty	As prognostic uncertainty is typical in neonatology, the conversation moves within a framework of uncertainty, which makes it difficult at times to convey clear information to parents, even though the conversation needs to be directional. However, this may include explicitly voicing this uncertainty to provide transparent information to parents.
E	Encounter in (triangular) Partnership	Respecting the unique triangular relationship between infant, parents and the whole treatment team must be assumed as a prerequisite for breaking bad news. This does not include the mere provision of information but an exchange.
O	Organisation and teamwork	Organisation and teamwork encompass dynamics within the care team that may affect staff-parent communication such as multiprofessional decision-making and jointly breaking bad news.
S	Situational stress	Situational stress recognizes general stressors of intensive care on parents, including unforeseen separation challenges concerning parents and infants.
P	Processuality	Breaking bad news discussions are to be understood as a process due to uncertainty and information gain during care. This principle of proscessuality can be supported by defining conversation routines (e.g., setting appointments, appointing fixed contact persons in the team…).
E	Emotional burden	Staff's emotional burden describes the strain placed on care teams by a breaking bad news process. Improving clinical processes, teaching, team communication, and collegial exchange can ease the burden and strengthen resilience to hold effective breaking bad news discussions.
A	Attention to individuality	Interindividual differences in the perception of events as well as individuals’ influence on the process itself and the interaction between subjects must be attended to. This concerns the individuality of the parents, but also of the professionals in terms of self-conception as a private or professional person.
K	Knowledge and experience	Experts’ knowledge and competencies substantially influence the conversation process. Soft skills thereby particularly increase motivation and lower resistance positions to enhance the tendency to cooperate in and around an organizational structure such as a NICU. For many decision-making processes, empirical values are an important tool on the basis of which clinical situations, parental information needs and conversation experience can be anticipated more accurately.

According to our study findings, three key dimensions of interactions should be respected when delivering bad news. There is a formal dimension, which includes the purely substantive meaning of the content of conversations, which clarifies medical aspects and is necessary to achieve informed consent. The second dimension stands for the newborn's parents, as message recipients, whose individual needs must be taken into account and for whom as little suffering as possible should be caused. Senders, the medical and nursing team, representing the third dimension, are the information source, but can also themselves be psychologically burdened by the message content and the course of conversation. From this, instead of the traditional linear relationship between sender, message and recipient, a reciprocal relationship can be derived. NEO-SPEAK is intended to consider all these dimensions and to present a specific framework for the challenges of difficult conversations in neonatology. Thereby NEO-SPEAK is not supposed to be a practical guideline in the sense of concrete instructions. Rather, it is about understanding which aspects need to be taken into account for the successful management of breaking bad news in neonatology.

The first three acronym components N-E-O are directly related to the specific care situation in neonatology: The special medical partnership and care context with the elements of (a) age-related diagnosis, prognosis, uncertainty, (b) encounter in partnership respecting the unique triangular relationship between newborns, parents and the whole treatment team and (c) the need for lively and intensive teamwork.

In neonatology, staff and parents frequently wrestle with difficult decisions under conditions of prognostic uncertainty with diverse long-term outcomes (6–9). This uncertainty is mainly due to the multiple factors associated with NICU mortality and dependence of prognosis on gestational age (41). However, in addition to this medical knowledge, a wealth of staff's experience and theoretical knowledge in the communication of bad news is therefore of advantage in order to be able to have sensitive and meaningful conversations with parents in which these uncertainties can also be openly addressed (33).

The triangular structure of relations and care procedures influence the decision-making process from individual autonomy to a *relational* concept of autonomy (42). Parents are in constant exchange of information with the entire team. Our study has shown when striving to improve the quality of communication with parents, it is not only the communication skills of individuals that are important, but also the communication within the team. The results suggest that good communicative teamwork in terms of decision-making and breaking bad news could also have a positive impact on communication with parents. In line with earlier studies informed by the parent perspective, a clear and honest information flow both within the multi-professional NICU team and between team members and the parents was recommended. This included congruent information from staff over time and, if possible, one professional serving as a continuous medical confident for families in charge of sharing information (36, 43).

The components of the framework, which are hidden behind the acronym part S-P-E-A-K, are important for breaking bad news in neonatology, but certainly not only relevant in this medical discipline (44). The individual points are not new and have already been elaborated as essential determinants in various communication guidelines (22, 45). The process nature of discussions over time, experience, attention to individuality, situational stress and emotional burden should be considered when delivering bad news in any setting, but may carry even more implications with the unique patient-parent-staff constellation in neonatology mentioned above. The delivery of distressing news is common in conversations between NICU staff and parents, but can have significant psychological effects on both parties (13, 15–17, 22, 31, 32). In this context, shared decision-making is key, which is promoted by tailored information but also by building trust. It seems self-evident that neonatologists must be considerate of parents' feelings. In the NICU setting, parents can feel overwhelmed or out of place (10), separated from their infant (15), and may experience difficulties in successfully growing into their parental role (11, 13, 17). However, our study also supports earlier research highlighting the need to strengthen staff's resilience as they face emotional challenges of dealing with critically ill neonates and their families (46). The NICU team is chronically exposed to the suffering of families faced with the illness or death of an infant, which can exacerbate an already emotionally difficult situation. It is well known that physicians in particular suffer considerably from this stress (47–49). This rather underestimated association when referring to competent physician-parent communication implies the necessity of measures such as supervision and peer consulting to improve psychosocial outcomes for NICU staff. Psychological stability and thus empathy on the part of the team members in turn facilitates the delivery of bad news.

First and foremost, our conceptual framework provides a snapshot of neonatologists' perspectives on the factors to consider for successful management of breaking bad news. Our mnemonic tool can help to better understand the framework for breaking bad news in neonatology and consider aspects that have received less focus. Based on our research, these aspects not only aim to improve the communication strategies of individual professionals, but also engage other patient care stakeholders within and outside the team, so that solid baselines for communication in the care network are developed. This includes optimizing hospital culture and policies to create fertile ground for communication.

### Strengths and limitations

Our qualitative study provides valuable insights into the views of seventeen experienced neonatologists on the framework of breaking bad news that could not have been captured by quantitative methods. By interviewing senior physicians, we were able to incorporate experiences related to internal influences and structures of daily clinical practice into our framework. With this study population, we gained an important and under-researched, but certainly limited perspective on terms and conditions for successful managing breaking bad news. Further qualitative and quantitative research including views of parents and the multi-professional NICU team will be necessary to provide a broader picture of the applicability and meaningfulness of the NEO-SPEAK framework. Particular attention should be paid to differences in appropriateness for end-of-life communication and conversations in the context of indefinite, complex long-term care, as this was not differentiated in our study. Moreover, some emerging aspects might only relate to hospital processes specific to the German context and are thus not internationally representative.

### Conclusion

The delivery of bad news, from the neonatologist's perspective, is subject to a framework that sets the conditions for successful managing breaking bad news in neonatology. From the results of our qualitative study, we were able to subsume this framework with its individual determinants under the acronym NEO-SPEAK. On the one hand, the framework shows which special aspects play a role in neonatology for the delivery of bad news, and on the other hand, it can help to identify and consider these aspects in clinical routine and training. While other communication tools focus exclusively on the recipients, NEO-SPEAK also considers the impact of conversations on the messenger and the importance of organizational aspects. Considering or reinforcing each NEO-SPEAK element when planning or delivering bad news can potentially impact communication with parents of critical ill premature and newborns infants and support the resilience of the caring team. Our framework can thus stimulate cultural changes in a facility to improve the experience of delivering bad news for parents, as well as for the caring team. Nevertheless, NEO-SPEAK should be evaluated for compatibility with the views of parents and interprofessional team members in a clinical context.

## Data Availability

The raw data supporting the conclusions of this article will be made available by the authors, without undue reservation.
